# Behavioral and molecular disruptions in honey bees induced by lithium chloride exposure

**DOI:** 10.1038/s41598-025-21359-x

**Published:** 2025-10-27

**Authors:** Mojtaba Esmaeily, Sedat Sevin, Tekalign Begna, Delgermaa Ulziibayar, Chuleui Jung

**Affiliations:** 1 Department of Plant Medicals, College of Life Sciences, Gyeongkuk National University, Andong, 36729 South Korea; 2 Department of Pharmacology and Toxicology, Faculty of Veterinary Medicine, Ankara University, Ankara, Turkey; 3 Department of Biology, National University of Mongolia, Ulaanbaatar, Mongolia

**Keywords:** *Apis mellifera*, *Varroa*, Acaricide, Survival, Aggressive behavior, Immune system, Ecophysiology, Entomology

## Abstract

Lithium chloride (LiCl) has emerged as a promising alternative to synthetic acaricides for controlling *Varroa destructor*, a major threat to honey bee health. However, its potential side effects on bees and their products require further investigation. This study aimed to assess the effects of LiCl on honey bee health, focusing on survival, behavior, and molecular responses. We investigated the effects of varying doses and feeding durations of LiCl on honey bee survival, aggression, stress- and immune-related gene expression, and recovery potential after treatment cessation. Our results show that LiCl feeding reduced honey bee survival in a dose-dependent manner, with higher concentrations leading to greater accumulation of lithium in bee tissues. Furthermore, LiCl suppressed aggression behavior and altered the gene expression related to honey bee health, such as vitellogenin, antimicrobial peptides, antioxidant enzymes, and heat shock proteins. The duration of LiCl feeding was a critical factor, as shorter feeding periods followed by recovery with control diets restored gene expression and survival rates to the levels of control groups. These findings highlight the importance of optimizing LiCl dosage and feeding duration to balance its *Varroa* controlling efficacy with honey bee safety, and further research is needed to ensure its long-term safety for colonies and ecosystems.

**Supplementary Information:**

The online version contains supplementary material available at 10.1038/s41598-025-21359-x.

## Introduction

Honey bees (*Apis mellifera*) are indispensable pollinators, playing a pivotal role in global food production and ecosystem sustainability^1^. By facilitating the reproduction of flowering plants, they enhance crop yields and support the biodiversity of wild plant species^1^.Their contributions to agricultural yields and biodiversity, however, are under significant threat due to global population declines^2^. Among the most severe threats to *A. mellifera* is *Varroa destructor* (Acari: Varroidae), a parasitic mite that is now nearly ubiquitous worldwide^3–7^. *V. destructor* is a leading cause of honey bee colony weakening and collapse^8–12^, as its parasitic feeding critically undermines bee health^13,14^. Furthermore, it serves as both a mechanical and biological vector for numerous pathogens, particularly viruses, exacerbating preexisting infections and amplifying colony losses^15,16^.

Over the past two decades, synthetic acaricides such as coumaphos, tau-fluvalinate, flumethrin, and amitraz have been widely used to control *V. destructor*^17,18^. However, their efficacy has been increasingly undermined by the development of mite resistance and the potential toxicity to non-target organisms, including honey bees^19–21^. These chemicals often leave residues in the hive, especially in wax and propolis, due to their lipophilic properties^22–24^. Given these concerns, natural compounds, including plant-based products and organic acids, have frequently been tested for their effectiveness against *V. destructor*^25–31^. However, none of these treatments, when used individually, guarantee sustained control, largely due to inconsistent or limited effectiveness. Moreover, these treatments can have adverse effects on honey bees^26,31^. As a result, the need for innovative approaches to control *Varroa* while safeguarding honey bee health is becoming increasingly urgent.

Lithium chloride (LiCl) was first identified as toxic to *V. destructor* by Ziegelmann et al.^32^, acting through a systemic mode of action via the honey bee’s body. Subsequent studies by Stanimirovic et al.^33^, Kolic et al.^34,35^, and Rein et al.^36^ demonstrated the efficacy of lithium salts in both cage trials and field experiments, achieving high mite mortality with minimal adverse effects on honey bee health. Erdem et al.^37^ further investigated the effects of lithium on honey bees. Their findings suggested that lithium may disrupt circadian rhythms and impair locomotor activity, which could potentially alter foraging behavior. Despite these behavioral concerns, LiCl has shown consistent efficacy when administered with appropriate dosage and treatment protocols, ensuring colony health and sustainability. Studies by Kolics et al.^35^ and Prešern et al.^38^ also reported that lithium residues in honey bee products are minimal and transient. Lithium levels in honey return to baseline within 16 days post-treatment, and contamination in bee bread and wax remains negligible. These findings suggest that lithium residues are unlikely to hinder the registration of LiCl as a veterinary acaricide.

Although Sevin et al.^39^ reported that lithium salts are generally safe for *A. mellifera*, they can affect behavioral traits and lifespan when used continuously. Given the promising efficacy of lithium salts against *V. destructor* and their minimal acute impact on honey bees, some studies have raised concerns about potential adverse effects on physiological and molecular aspects in insects. For instance, Kasuya et al.^40^ demonstrated that lithium can significantly alter gene expression in *D. melanogaster*, affecting genes involved in amino acid transport and metabolism, detoxification, stress response, and neurological functions. Furthermore, Wojciak et al.^41^ found that lithium ingestion in Madagascar cockroaches led to notable morphological changes in their neuroendocrine systems, causing significant enlargement of neuroendocrine structures. In honey bees, disruptions in the expression of key genes related to essential biological functions may signal adverse effects of lithium. Vitellogenin, a multifunctional protein, is crucial for reproduction, immunity, and oxidative stress regulation, with altered expression linked to impairments in these pathways^42,43^. Antimicrobial peptides (AMPs) are sensitive indicators of immune modulation under environmental stressors^44,45^. Additionally, genes encoding antioxidant enzymes such as superoxide dismutase (SOD) and catalase (CAT), along with heat shock proteins (HSPs), reflect responses to oxidative stress and efforts to maintain cellular homeostasis^46,47^. Examining these genes provides a comprehensive framework for understanding lithium’s effects on honey bee reproduction, immunity, stress responses, and oxidative balance, offering critical insights into its broader implications for colony health.

This study aims to evaluate the effects of LiCl on honey bees, focusing on survival, behavioral responses, and molecular changes. By analyzing the expression of key genes related to immunity, stress, and antioxidant defenses, along with recovery potential post-treatment, this research provides novel insights into the safety and long-term implications of LiCl use in honey bee colonies.

## Results

### Dose-dependent effect of LiCl on honey bee survival

**Fig. 1:**
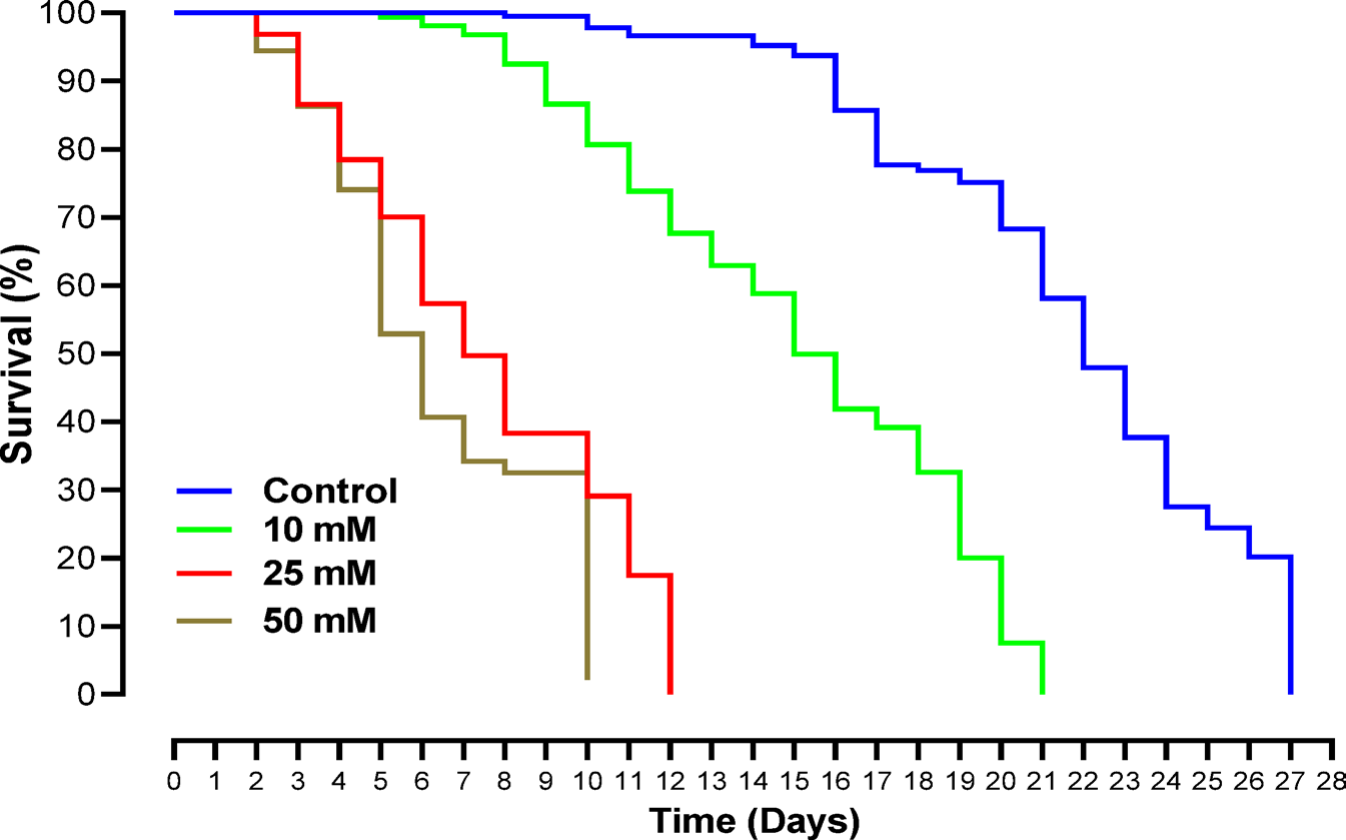
Risk assessment of LiCl in *A. mellifera*. Survival analysis of *A. mellifera* in response to different concentrations (10, 25 and 50 mM) of LiCl. Each treatment was replicated 10 times with 10 bees in each (*n* = 100), with mortality rates recorded till they all died. In the control group, bees were fed by 50% sucrose.

The toxicity of different concentrations of LiCl (10, 25, and 50 mM) on honey bees was evaluated through lifetime feeding assays. Kaplan-Meier survival analysis demonstrated that LiCl significantly reduced survival rates in a dose-dependent manner (Fig. 1). A log-rank test indicated significant differences among the groups (χ² = 102.36, df = 3, *p* < 0.0001). The 50 mM treatment caused the highest mortality, with all bees dying within 10 days, while the 25 mM treatment resulted in complete mortality by day 12. In contrast, bees in the 10 mM treatment exhibited no mortality during the first 7 days, but their survival rate began to decline afterward, reducing their lifespan by approximately one week compared to the control. Mortality analysis at 7 days after treatment further highlighted these differences, showing that bees exposed to 25 and 50 mM LiCl experienced 58% and 74% mortality, respectively, while no significant mortality was observed in the control or 10 mM treatment groups (Fig. 1).

### Lithium accumulation in honey bee bodies

**Fig. 2:**
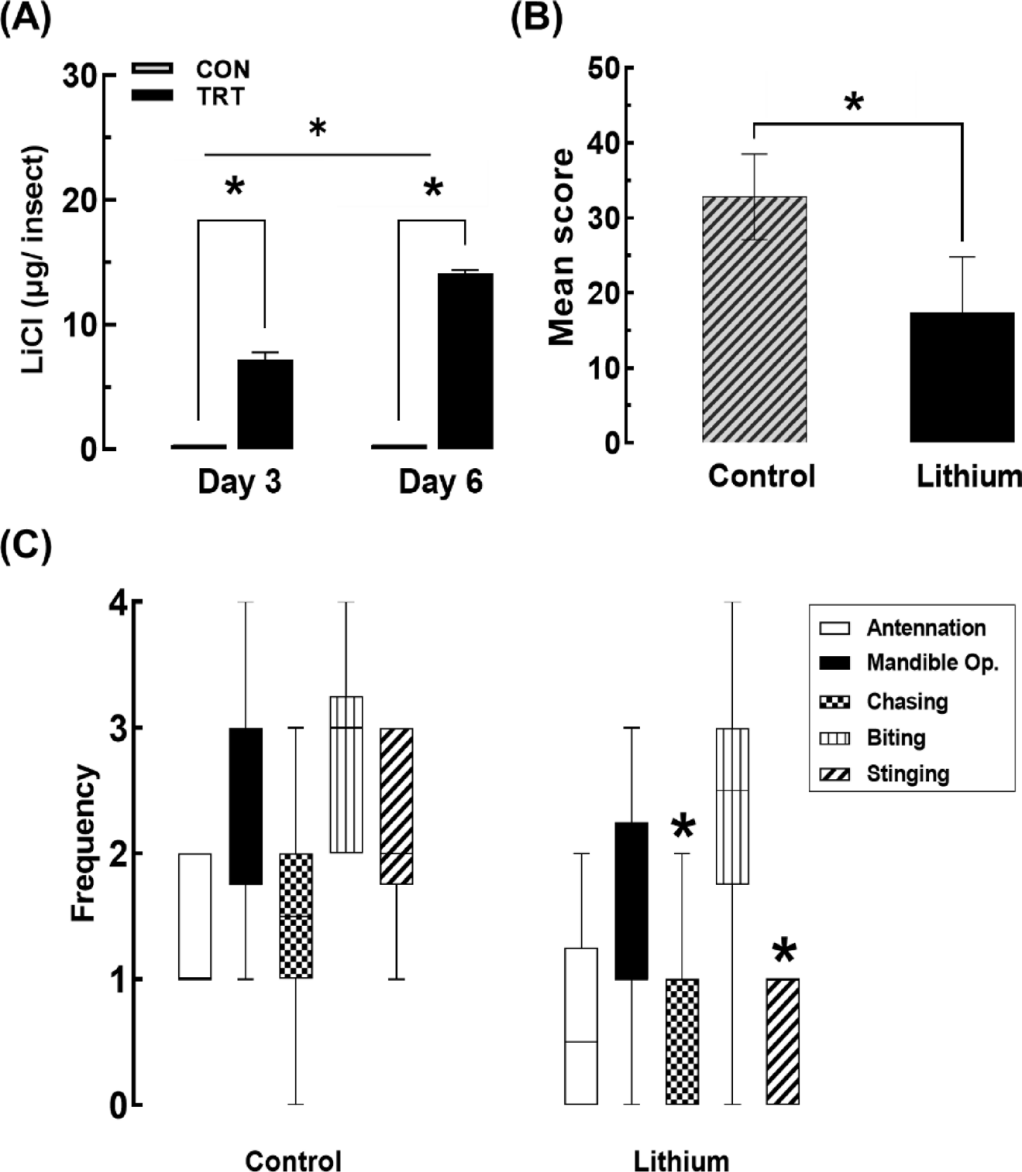
Lithium accumulation in *A. mellifera* and its impact on aggressive behavior. (**A**) Lithium concentration in *A. mellifera* bodies after feeding with 10 mM LiCl for 3 and 6 days, detected using ICP analysis (Mean ± SE). Each measurement was duplicated with three independent biological replicates (*N* = 3). For each replicate, 50 bees were pooled and processed as a single composite sample. Asterisks (*) indicate significant differences compared to the control group at a Type I error rate of 0.05 (Tukey’s HSD test). (**B**) Total aggression scores (Mean ± SE) in control and LiCl-fed groups, measured during an intruder assay conducted on the 7th day. Each measurement was duplicated with ten independent biological replicates (*N* = 10), with 10 bees per replicate (*n* = 10). Asterisk indicates significant differences compared to the control group at a Type I error rate of 0.05 (Tukey’s HSD test). (**C**) Frequency of individual aggressive behaviors (antennation, mandible opening, chasing, biting, and stinging) (Mean ± SE) in control and LiCl-fed bees during the intruder assay. Asterisks indicate statistically significant differences compared to the control group (*p* < 0.05, Mann–Whitney U test). Each measurement was duplicated with ten independent biological replicates (*N* = 10), with 10 bees per replicate (*n* = 10).

The results, as shown in Fig. 2A, reveal a significant increase in lithium concentration in the treated group compared to the control group on day 3 (t = -10.98, *p* = 0.00039). Furthermore, lithium levels were significantly higher on day 6 than on day 3 in the treated bees (t = -10.55, *p* = 0.00046), indicating a time-dependent accumulation of lithium in honey bee tissues.

### Impact of lithium on aggressive behavior

Behavioral assays were conducted to evaluate the impact of lithium exposure on the aggressive behavior of worker bees. As shown in Fig. 2B and detailed in supplementary Table S3, the mean aggression score was significantly lower in the LiCl-fed group compared to the control group, indicating that lithium exposure suppressed overall aggression levels in *A. mellifera*.

Figure 2C provides a detailed analysis of individual aggressive behaviors. Bees in the LiCl-treated group exhibited a significant reduction in the frequency of stinging and chasing behaviors (*p* < 0.05), which are considered high-severity actions in the aggression scale. While reductions were also observed in antennation, mandible opening, and biting, these differences were not statistically significant. These findings suggest that lithium accumulation reduces both the intensity and frequency of aggressive responses in *A. mellifera*.

### Feeding-time-dependent effect of LiCl on honey bee survival and *Am-Vg* expression

**Fig. 3:**
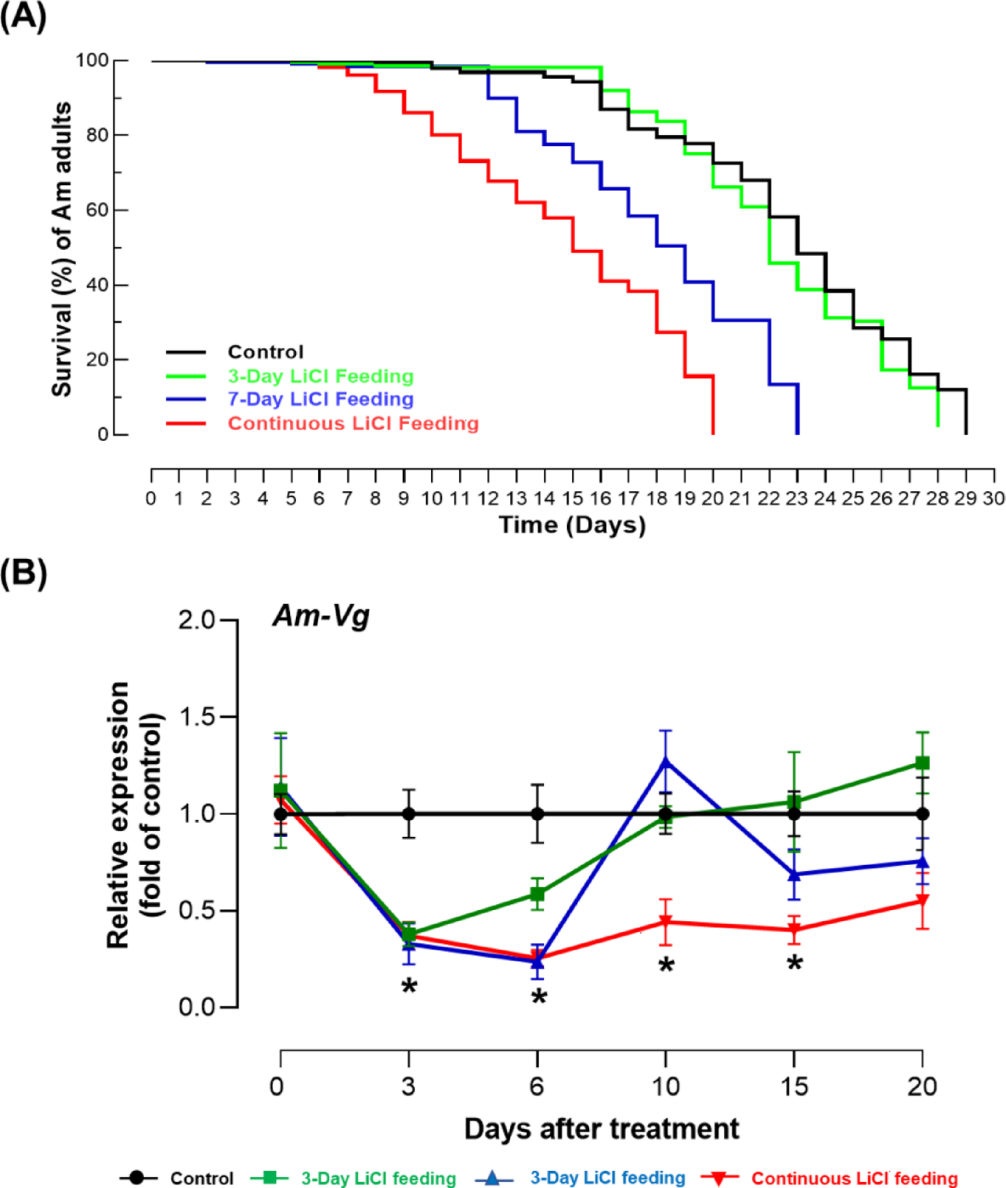
Effects of LiCl feeding on survival and *Am-Vg* expression in *A. mellifera*. (**A**) Kaplan-Meier survival analysis of *A. mellifera* under different LiCl feeding durations. Four groups were tested: Control (fed with 50% sucrose solution), 3-Day LiCl Feeding (fed with 10 mM LiCl for 3 days, then switched to 50% sucrose), 6-Day LiCl Feeding (fed with 10 mM LiCl for 6 days, then switched to 50% sucrose), and Continuous LiCl feeding (fed with 10 mM LiCl until death). Each treatment included 10 replicates (*N* = 10) with 10 bees per replicate (*n* = 100), with mortality recorded daily until all bees had died. (**B**) *Am-Vg* expression in *A. mellifera* across all groups (Control, 3-Day, 6-Day, and Continuous LiCl feeding) in response to 10 mM LiCl feeding, measured at 3, 6, 10, 15, and 20 days after the initiation of the experiment (Mean ± SE). Expression analysis was performed with three independent samples per time point (*N* = 3). Asterisks indicate significant differences compared to the control group at a Type I error rate of 0.05 (Tukey’s HSD test).

To evaluate the effect of LiCl feeding duration on honey bee survival, four treatment groups were analyzed (Fig. 3A). Bees continuously fed 10 mM LiCl exhibited the shortest lifespan, with a median survival of 15 days. Those fed LiCl for 6 days and then switched to 50% sucrose solution showed improved survival compared to continuous feeding, with a median survival of 18 days. Conversely, bees fed LiCl for only 3 days followed by sucrose exhibited the longest survival, closely resembling the control group fed sucrose throughout their lives. A log-rank (Mantel–Cox) test revealed statistically significant differences among the treatment groups (χ² = 102.36, df = 3, *p* < 0.0001). Continuous LiCl feeding resulted in significantly lower survival compared to all other groups (*p* < 0.001), while 6-day LiCl exposure led to intermediate survival, significantly better than continuous feeding (*p* < 0.01) but lower than the control (*p* < 0.05). The 3-day LiCl feeding group showed no significant difference from the control (*p* > 0.05), indicating that reducing the duration of LiCl exposure alleviates its toxic effects on honey bee survival.

On the other hand, the expression pattern of *Am-Vg*, as a key biomarker of honey bee health and longevity, was analyzed after 3, 6, 10, 15, and 20 days across all feeding regimes (Fig. 3B).

A two-way ANOVA revealed significant effects of feeding duration (*p* < 0.0001), treatment (*p* < 0.0001), and their interaction (*p* < 0.0001) on *Am-Vg* expression levels, indicating that the impact of LiCl on *Am-Vg* expression varied over time and treatment combinations.

Bees continuously fed 10 mM LiCl exhibited a sharp decline in *Am-Vg* expression from day 10 onward, which correlated with increased mortality rates. In contrast, bees that received LiCl for 3 days and were then switched to sucrose showed *Am-Vg* expression patterns similar to the control group throughout the time course (*p* > 0.05), while the 6-day group showed intermediate recovery. These results highlight that both the duration of exposure and the treatment itself significantly modulate *Am-Vg* expression, and that early withdrawal of LiCl can help preserve gene expression and physiological function.

### Effect of LiCl on antimicrobial peptides (AMP) gene expression

To evaluate the effect of LiCl on the expression of AMP genes, including defensin (*Am-Def*), hymenoptaecin (*Am-Hym*), abaecin (*Am-Aba*), and apidaecin (*Am-Api*), honey bees were fed 10 mM LiCl for varying durations, and gene expression was analyzed on days 0, 3, 6, 10, 15, and 20 after treatment. This experiment not only assessed the effect of LiCl on AMP expression but also examined its recovery patterns under different feeding durations.

**Fig. 4:**
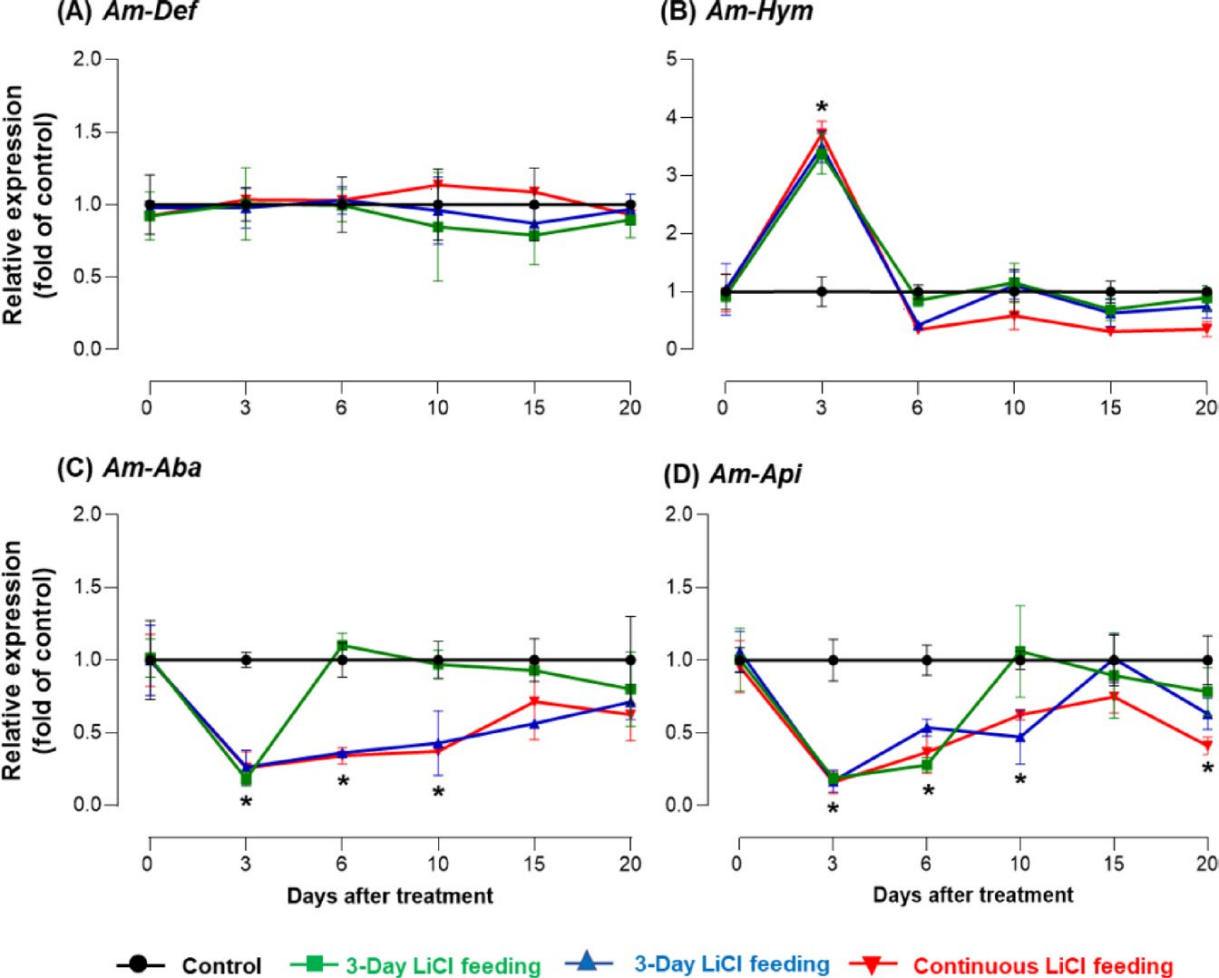
Effects of LiCl feeding on immune-related gene expression in *A. mellifera.* Expression of hymenoptaecin (*Am-Hym*) (**A**), defensin (*Am-Def*) (**B**), abaecin (*Am-Aba*) (**C**), and apidaecin (*Am-Api*) (**D**) in *A. mellifera* across all groups (Control, 3-Day, 6-Day, and Continuous LiCl Feeding) in response to 10 mM LiCl feeding, measured at 3, 6, 10, 15, and 20 days after the initiation of the experiment (Mean ± SE). Expression analysis was performed with three independent samples per time point (*N* = 3). Asterisks indicate significant differences compared to the control group at a Type I error rate of 0.05 (Tukey’s HSD test).

A two-way mixed-effects model revealed significant main effects of feeding treatment, time, and their interaction on most AMP genes. For *Am-Def*, no statistically significant interaction or main effects were found (Treatment: F = 1.12, *p* = 0.35; Time: F = 1.23, *p* = 0.29; Interaction: F = 1.11, *p* = 0.37), indicating that LiCl exposure did not significantly affect *Am-Def* expression throughout the experiment (Fig. 4A). In contrast, *Am-Hym* expression was significantly influenced by both treatment and time (Treatment: F = 136.4, *p* < 0.0001; Time: F = 74.2, *p* < 0.0001; Interaction: F = 51.6, *p* < 0.0001). Expression levels sharply increased after 3 days of LiCl exposure, especially in the continuous feeding group, and then gradually declined over time (Fig. 4B). This pattern indicates a strong early immune activation, followed by a suppression or adaptation phase. For *Am-Aba*, there was a significant reduction in expression in response to LiCl across time (Treatment: F = 101.9, *p* < 0.0001; Time: F = 33.6, *p* < 0.0001; Interaction: F = 27.4, *p* < 0.0001). Suppression began as early as 3 days post-treatment and remained consistent, especially in the continuous feeding group (Fig. 4C). Similarly, *Am-Api* expression showed a significant decline following LiCl exposure, with strong effects of treatment, time, and their interaction (Treatment: F = 142.5, p *<* 0.0001; Time: F = 41.8, *p* < 0.0001; Interaction: F = 36.7, *p* < 0.0001) (Fig. 4D). Expression levels were lowest in the continuous group and began recovering only in the 3-day feeding group after day 6, suggesting partial reversibility upon LiCl withdrawal. These findings confirm that LiCl has a differential and time-dependent effect on AMP gene expression, with *Am-Hym* responding biphasically, while *Am-Aba* and *Am-Api* are consistently downregulated. The results also emphasize that short-term LiCl exposure allows for recovery, whereas prolonged feeding leads to prolonged immunosuppression.

### Effect of LiCl on stress-related genes expression

**Fig. 5:**
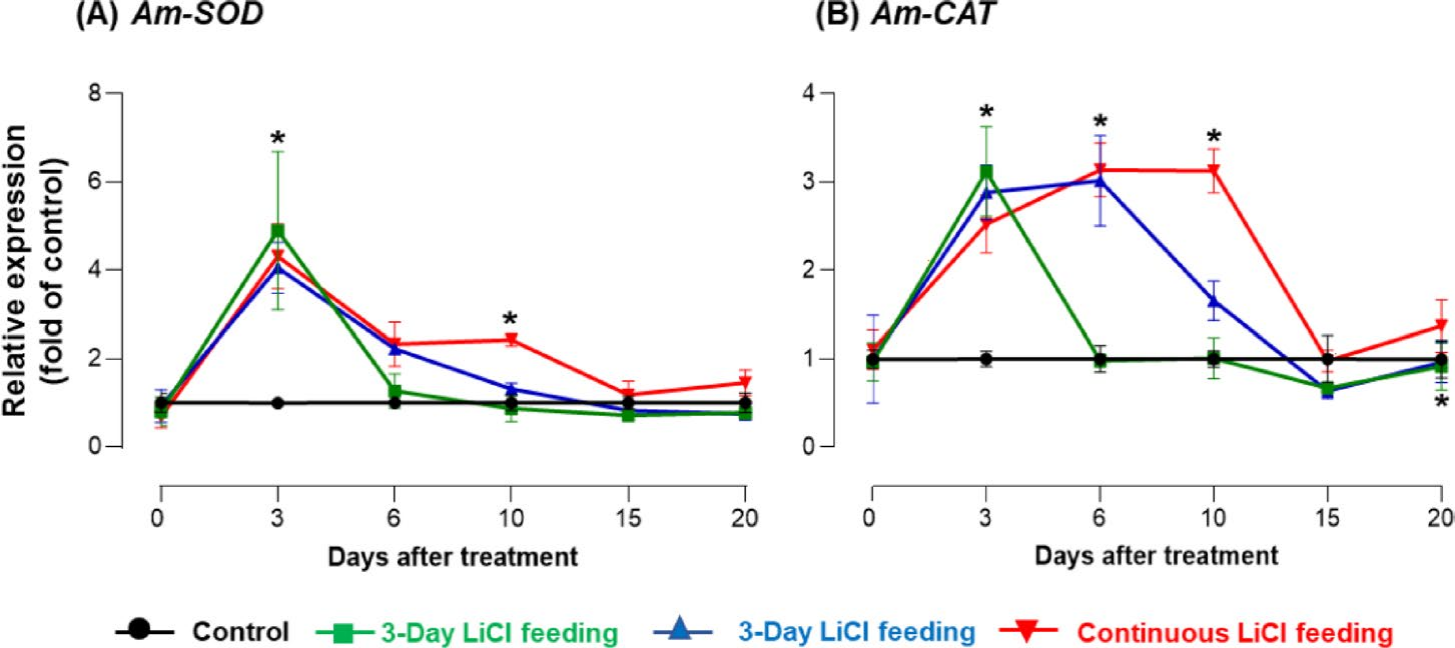
Effects of LiCl feeding on oxidative stress-related gene expression in *A. mellifera*. Expression of catalase (*Am-CAT*) (**A**) and superoxide dismutase (*Am-SOD*) (**B**) in *A. mellifera* across all groups (Control, 3-Day, 6-Day, and Continuous LiCl Feeding) in response to 10 mM LiCl feeding, measured at 3, 6, 10, 15, and 20 days after the initiation of the experiment (Mean ± SE). Expression analysis was performed with three independent samples per time point (*N* = 3). Asterisks indicate significant differences compared to the control group at a Type I error rate of 0.05 (Tukey’s HSD test).

LiCl treatment significantly upregulated the expression of *Am-CAT* (F = 29.18, *p* < 0.0001) and *Am-SOD* (F = 23.65, *p* < 0.0001) in honey bees, with distinct patterns across feeding treatments and time points (Fig. 5). A two-way ANOVA revealed significant main effects of treatment and time, as well as a significant interaction between treatment and time for both genes (*Am-CAT*: F_treatment = 29.18, F_time = 63.62, F_interaction = 11.49, all *p* < 0.0001; *Am-SOD*: F_treatment = 23.65, F_time = 33.25, F_interaction = 4.88, all *p* < 0.0001). In the 3-day LiCl feeding group, antioxidant gene expression increased significantly by day 3 but returned to control levels at later time points, indicating recovery from oxidative stress. In contrast, the 6-day and continuous LiCl feeding groups showed a more prolonged upregulation, especially notable for *Am-CAT* (Fig. 5B), with expression levels remaining significantly higher than control up to day 15. These findings confirm that LiCl induces oxidative stress in a time-dependent manner, and that short-term exposure allows partial or full recovery, whereas longer-term exposure leads to sustained antioxidant gene expression.

**Fig. 6:**
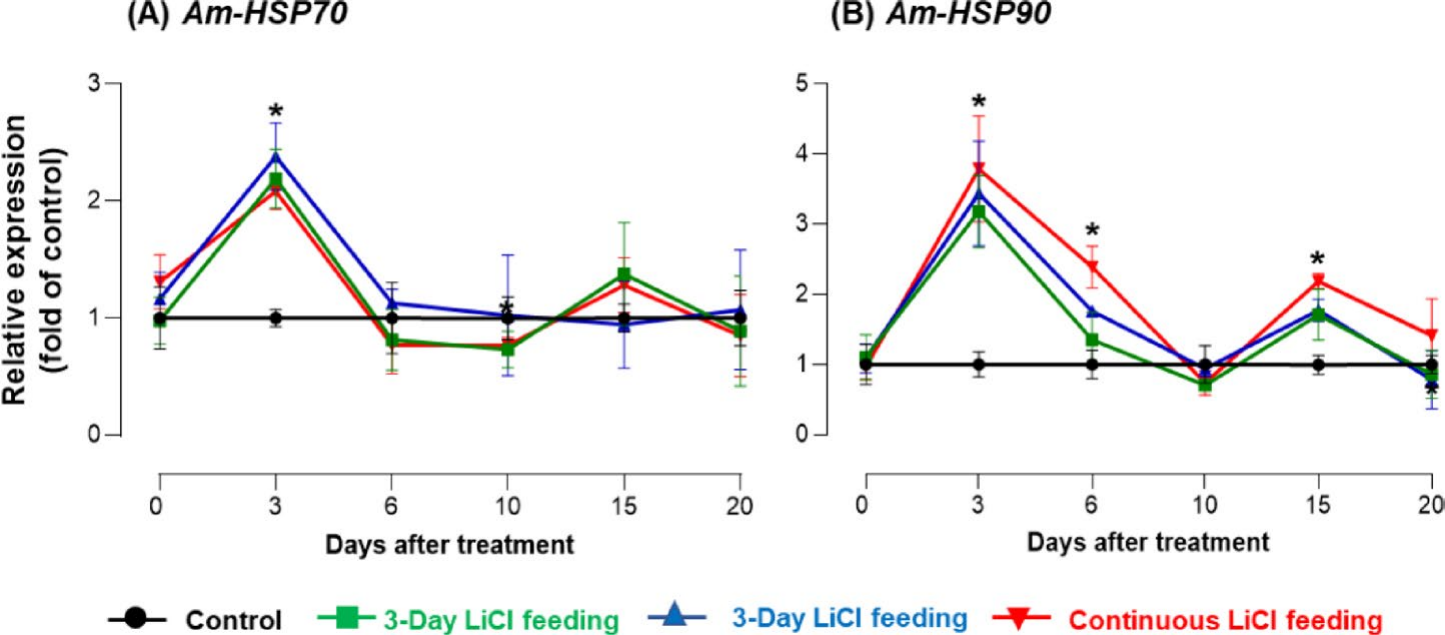
Effects of LiCl feeding on heat shock protein gene expression in *A. mellifera*. Expression of HSP70 (*Am-HSP70*) (**A**) and HSP90 (*Am-HSP90*) (**B**) in *A. mellifera* across all groups (Control, 3-Day, 6-Day, and Continuous LiCl Feeding) in response to 10 mM LiCl feeding, measured at 3, 6, 10, 15, and 20 days after the initiation of the experiment (Mean ± SE). Expression analysis was performed with three independent samples per time point (*N* = 3). Asterisks indicate significant differences compared to the control group at a Type I error rate of 0.05 (Tukey’s HSD test).

Moreover, as shown in Fig. 6A, *Am-HSP70* expression was significantly affected by both treatment (F = 2.99, *p* = 0.0402) and time (F = 21.31, *p* < 0.0001), with a significant interaction effect (F = 3.09, *p* = 0.0015). The expression of *Am-HSP70* increased sharply after 3 days of LiCl exposure in all treatment groups, particularly at 50 mM concentration. However, this elevation was transient, as expression levels gradually returned to baseline or control values by day 10, suggesting that the impact of LiCl on *Am-HSP70* expression is temporary and reversible, especially after short-term exposure. Conversely, as shown in Fig. 6B, *Am-HSP90* expression showed a much stronger and sustained response, with significant effects of treatment (F = 13.90, *p* < 0.00001), time (F = 28.67, *p* < 0.00001), and interaction (F = 4.86, *p* = 0.0004). Expression levels of *Am-HSP90* increased significantly after 3 days of LiCl exposure across all doses, and remained elevated particularly in the 6-day and continuous feeding groups, reflecting persistent cellular stress. Only in the 3-day feeding group did expression levels gradually return to those of the control, suggesting partial recovery. These results confirm that LiCl-induced stress activates *Am-HSP90* more robustly and persistently than *Am-HSP70*, especially under prolonged exposure conditions.

## Discussion

*Varroa destructor* is currently one of the most severe threats to honey bees, often leading to colony weakening and collapse, which critically threatens the health and sustainability of bee colonies^9–11^. While synthetic acaricides are commonly used, there is a need for alternative solutions that balance effective mite control with bee health protection. Lithium salts, especially LiCl, have shown promising acaricidal effects^32,33,39,48^ and have been validated in field conditions^34,36,49^. However, their potential side effects on bees and their products require further investigation before lithium can be adopted as a reliable *Varroa* control method.

Our findings indicate that LiCl has significant adverse effects on honey bees, particularly when it is continuously present in their diet, reducing survival rates in a dose-dependent manner. Even at a concentration of 10 mM, LiCl substantially increased mortality and shortened worker bees’ lifespans. These results are consistent with previous studies^32,50^. Lithium’s accumulation within honey bee bodies and honey is another critical factor contributing to its negative impact. Consistent with earlier researches^32,35,38,50^, our results showed a time-dependent increase in lithium concentrations in honey bees, which can impair their fitness and survival. However, differences in survival and lithium accumulation across studies may reflect variations in experimental settings. For instance, Rein et al.^51^ conducted whole-hive trials in field conditions, where social buffering, spatial dilution, and complex trophallactic interactions may mitigate the effective exposure of individuals to lithium. In contrast, our use of caged bees in controlled laboratory conditions ensured uniform and continuous exposure, potentially amplifying LiCl’s effects on mortality and tissue retention. These methodological distinctions should be considered when comparing results across studies. Although Rein et al.^51^ reported minimal lithium accumulation (0.52 mg/kg) in the hypopharyngeal glands, with limited risk to queens and early instar larvae under continuous 50 mM LiCl treatment for 7 days, concerns remain regarding the physiological and behavioral effects of even trace amounts of lithium. Lithium may disrupt neurological pathways, altering honey bee behavior. For example, Hurst et al.^52^ demonstrated that lithium induces malaise behavior. Additionally, studies by Erdem et al.^37^ and Sevin et al.^39^ highlighted lithium’s effects on locomotor activity, and similarly we revealed that LiCl significantly suppressed aggression levels in honey bee. Aggression is critical for colony defense, as it enables bees to protect their hive from intruders and potential threats. Reduced aggression could compromise the colony’s ability to safeguard its members and resources, ultimately weakening overall colony health and resilience^53,54^. These findings emphasize the need to thoroughly evaluate lithium’s long-term effects on both individual bees and colony dynamics. These findings highlight the need to carefully evaluate lithium’s potential impacts on bee health, particularly in relation to its accumulation over time.

Our findings indicate that compared to higher concentrations,10 mM LiCl has the least adverse effect on honey bee survival, while still being effective in controlling *Varroa* mites, as demonstrated in previous studies^32,55^. Building upon this, our additional experiments showed that while 10 mM LiCl negatively affects honey bee survival, implementing short-term feeding with lithium salt followed by switching the diet back to control not only successfully reduces mites^32,33^ but also causes less adverse effects on bee survival. These results emphasize that the duration of LiCl exposure plays a crucial role in achieving effective mite control with minimal harm to honey bees.

Our results show that LiCl treatment reduces *Am-Vg* expression in honey bees after 3 days. While limited research on lithium salts’ effects on *Am-Vg* exists, our findings contrast with Jovanovic et al.^55^, who reported increased *Am-Vg* expression with lithium citrate in the *Varroa* infected bees. However, Inokuchi et al.^56^ found that LiCl decreases *Am-Vg* expression in the nematode *Caenorhabditis elegans*, which aligns with our results. Additionally, several acaricides and insecticides, including those mentioned by Liu et al.^57^, Chaimanee et al.^58^ Yao et al.^59^ and Zhou et al.^60^, have been shown to reduce *vitellogenin* expression, further supporting our findings. Since vitellogenin regulates honey bee lifespan, its downregulation may explain the observed shortened lifespans following LiCl treatment.

Although our data show reduced *Am-Vg* expression following LiCl treatment, it remains unclear whether this is a direct transcriptional effect or a secondary consequence of systemic toxicity. Given Vg’s known roles in stress and longevity regulation, the observed downregulation may reflect physiological stress induced by LiCl exposure. However, bees fed LiCl for a short-term followed by sucrose were able to restore *Am-Vg* levels to those of the control group, while other treatments remained significantly lower. These findings suggest that short-term LiCl feeding preserves survival rates and enables physiological recovery, emphasizing LiCl’s potential as a targeted *Varroa* treatment when used appropriately.

Several studies have shown that exposure to metal ions can disrupt immune homeostasis in honey bees. In our study, LiCl treatment downregulated the expression of *Am-Aba* and *Am-Api*, had no significant effect on *Am-Def*, and upregulated *Am-Hym* after 3 days. This biphasic response suggests differential sensitivity of antimicrobial peptide (AMP) genes to lithium exposure. Similar patterns have been observed with other metals. For instance, cadmium (Cd) exposure in *Apis cerana* suppressed *abaecin* and *apidaecin* expression, disrupted immune signaling, and increased mortality^61^. Furthermore, Polykretis et al.^62^ demonstrated that cadmium exposure significantly suppressed immune competence in *Apis mellifera*, with observable reductions in immune responses after just 3 days. This immunosuppression was supported by both immune challenge assays and structural analysis of fat body cells, indicating that Cd contamination can impair bees’ ability to resist pathogens. Conversely, the downregulation of *Am-Aba* and *Am-Api* in our study differs from findings by Jovanovic et al.^55^, who reported upregulation of these AMPs following treatment with lithium citrate. This discrepancy may stem from differences in lithium salt form, concentration, exposure duration, or experimental conditions.

On the other hand, recent studies have shown that stressful conditions lead to differential regulation of genes involved in antioxidant enzymes and heat shock proteins. Our results indicate that LiCl treatment upregulated oxidative stress-related genes, such as catalase and superoxide dismutase, suggesting an activation of antioxidant defenses in response to cellular stress. This likely reflects the production of reactive oxygen species (ROS), a known effect of lithium salts and chemical stressors^63–65^. To counteract these detrimental effects, organisms, including honey bees, activate antioxidant defense systems that has been also observed in response to lithium citrate^55^ and other stressors, such as pesticides^66–68^. These parallels suggest that LiCl may induce oxidative stress in a manner akin to pesticides, triggering a protective transcriptional response in honey bees. In recent years, numerous studies have investigated the gene expression profiles of heat shock proteins (HSPs) under various stress conditions^46,69–71^. Similarly, LiCl treatment upregulated *Am-HSP70* and *Am-HSP90*, indicating a stress-induced response and highlighting their role in cytoprotection and protein homeostasis, consistent with findings from other studies on environmental and chemical stressors^72^. The upregulation of *Am-HSP70* and *Am-HSP90* indicates that LiCl induces oxidative stress, triggering protective pathways to mitigate cellular damage and maintain protein quality. This aligns with evidence of HSP responses under various environmental and chemical stressors, highlighting their role in stress resilience and cellular defense.

Recovery of physiological and molecular processes after stress is critical for the survival and health of organisms. In our study, honey bees fed with LiCl for three days followed by a return to normal sucrose feeding exhibited a remarkable recovery in the expression of key genes involved in immunity (AMPs), antioxidant defense, and stress response (HSPs). This recovery underscores the transient nature of LiCl-induced disruptions and highlights the resilience of honey bees when the stressor is removed promptly. In contrast, bees subjected to six days of LiCl feeding or continuous exposure experienced prolonged disruptions in gene expression and reduced survival rates, indicating that the duration of LiCl exposure is pivotal. These findings suggest that short-term LiCl exposure allows bees to restore homeostasis as seen in other organisms^73,74^, likely through mechanisms like protein repair, antioxidant activation, and immune balance. Optimizing LiCl treatment duration could enable its effective use against *Varroa* mites while minimizing adverse effects on honey bees. Although field-level implementation of short-term LiCl treatments presents clear logistical challenges, our findings offer a proof-of-concept under controlled laboratory conditions. Future work should explore the development of practical delivery systems—such as controlled feeders or slow-release formulations—that enable time-restricted exposure while maintaining *Varroa* control efficiency.

In conclusion, this study investigated LiCl, a compound recently proposed as a potential acaricide for controlling *Varroa* mites in beekeeping. While LiCl demonstrates efficacy against mites, our findings reveal its adverse effects on honey bees, especially at higher concentrations. Reducing the concentration to 10 mM mitigated these harmful effects. Although this study did not directly assess mite control at this concentration, previous reports have suggested that 10 mM LiCl may still offer some acaricidal activity, highlighting its potential for balancing mite control and bee safety. Additionally, the duration of exposure proved critical; continuous feeding led to increased mortality and disrupted key physiological and behavioral functions. In contrast, limiting the exposure to three days minimized these negative effects, allowing bees to recover quickly, with gene expression and survival rates returning to near-normal levels. Despite these promising results, further research is necessary to evaluate the long-term impacts of LiCl on different honey bee castes, colony dynamics, and its broader ecological consequences. Optimizing LiCl usage could make it a valuable tool for *Varroa* management, but its safety must be ensured to protect honey bee health and colony sustainability.

## Experimental procedures

### Insects

Newly emerged bees were obtained from capped brood frames collected from healthy colonies in the apiary of the Insect Ecology Laboratory, Gyeongkuk National University, Andong, South Korea. The frames were incubated at 33 ± 0.1 °C and 65 ± 5% RH, and bees that emerged within 12 h were collected and used for the experiments. For the assessment of LiCl on *A. mellifera*, 10 newly emerged bees were placed in bee-rearing cages (100 × 40 mm, SPL Life Sciences, Pocheon, Korea) following a 24-hour adaptation period. The bees were reared under controlled conditions at a temperature of 33 ± 0.1 °C and relative humidity of 65 ± 5%. In the normal condition (without any treatment), bees were provided with pollen patty (Pyung Hwa industry, Gongju, South Korea) and a 50% sucrose solution (W/V), which was refreshed daily.

### Survival and longevity experiment

To evaluate the effects of LiCl on honey bee survival, two separate bioassays were conducted using the bees described above.

In the first experiment, newly emerged bees were fed a 50% sucrose solution (W/V) containing one of three concentrations of LiCl (10, 25, and 50 mM) (purchased from Sigma Aldrich Korea, Seoul, South Korea).

In the second experiment, newly emerged bees were fed 10 mM LiCl, with feeding duration differing among three treatment groups:

1. Bees were fed with 10 mM LiCl for 3 days, then switched to 50% sucrose solution for the remainder of their lives.
2. Bees were fed with 10 mM LiCl for 6 days, then switched to 50% sucrose solution.
3. Bees were fed with 10 mM LiCl continuously until death.

For both experiments, the control groups were fed 50% sucrose solution throughout their lifetimes. Treatment solutions were refreshed daily, and mortality was recorded daily until all bees had died. Each group included 10 replicates, with 10 bees per replicate. The data from both experiments were analyzed to evaluate the impact of LiCl concentration and feeding duration on honey bee survival.

### Insect sample preparation for quantification of LiCl

To measure LiCl accumulation in the *A. mellifera* body, bees were fed 10 mM LiCl. Samples were collected after 3 and 6 days exposure. Each measurement was duplicated with three independent biological replicates, with 50 bees per replicate. The bees from each treatment group and the control group were collected, and 1 g of sample per treatment were weighed using an analytical balance (ES 225SM-DR, Precisa, Dietikon, ZH, Switzerland). The samples were placed in 50 mL glass beakers and dried at 50 °C to a constant weight in an electric drying cabinet. Drying continued until the samples reached a stable weight. Dried samples were then wet-digested directly in the same beakers to minimize cross-contamination. A digestion mixture consisting of 4.0 mL of 65% (w/w) nitric acid (HNO_3_; reagent grade, Scharlau, Germany) and 1.0 mL of 30% (w/w) hydrogen peroxide (H_2_O_2_; reagent grade, Merck, Kenilworth, NJ, USA) was added. The digested solutions were quantitatively transferred to calibrated plastic centrifuge tubes and diluted to a final volume of 10 mL with ultrapure water (Synergy UV, Sigma-Aldrich, St. Louis, MO, USA). Prepared solutions were stored at room temperature until elemental analysis. All glassware used in the procedure was decontaminated by immersion in a 1:5 HNO_3_ solution for 24 h, followed by thorough rinsing with deionized water and air-dried to prevent contamination.

### Inductively coupled plasma (ICP) analysis

The lithium content in the prepared samples was quantified using microwave plasma atomic emission spectrometry (MP-AES 4200, Agilent Technologies, Santa Clara, CA, USA). Nitrogen gas, continuously supplied by a nitrogen generator (4107, Agilent Technologies), was used as the plasma gas. The MP-AES system was operated with a vertical torch alignment and an axial observation position. Sample solutions, along with standards, were introduced via an autosampler (SPS, Agilent Technologies), with a 30-second rinse using 0.1 M HNO_3_ (prepared in ultrapure water) between each measurement. Operating conditions and measurement parameters for the MP-AES are provided in Table S1. A lithium standard stock solution (1000 mg L⁻¹; Scharlau, Germany) was used to prepare a 5-point calibration series. The limit of detection (LOD) for lithium was determined to be 0.3246 µg kg⁻¹ at the selected wavelength of 610.365 nm. The LOD was calculated using the formula: LOD = (3 × s) / S where s is the standard deviation of 15 blank samples, and S is the slope of the calibration curve. Results from the elemental analysis were reported on a dry mass basis.

### Behavioral assay

Newly emerged adult worker bees were collected from Gyeongkuk National University apiary, Andong, South Korea. Frames of comb containing pupae showing signs of emergence were taken from colonies headed by naturally mated queens and incubated at 33 ± 1 °C and 65 ± 1% RH. The bees were individually marked with different colors and placed in groups of 10 inside vertically oriented Petri dishes (90 × 20 mm) lined with a beeswax foundation plate to mimic hive conditions (Fig. S1)^75^ Bees were fed either a 50% sugar solution (control group) or a 10 mM Lithium Chloride (LiCl)-containing sugar solution (treatment group). Pollen patties were provided as supplementary food.

To evaluate the effect of LiCl treatment on aggression, an intruder assay was conducted on the seventh day^76,77^. A forager from a different colony was introduced into each group, and the behaviors of the bees were recorded for 5 min using a digital video camera. Aggressive actions—antennation, mandible opening, chasing, biting, and stinging—were classified and scored based on severity, with scores ranging from 1 (least aggressive) to 5 (most aggressive), following a standardized ethogram previously used in honey bee aggression studies. Video recordings were analyzed manually by three trained observers who were blinded to the treatment groups. Each observer independently scored the videos, and the mean of the three scores for each behavior was used for statistical analysis. For each group, a total aggression score was calculated by multiplying the number of occurrences of each behavior by its assigned severity score, and summing across all behaviors. This final score reflected both the frequency and intensity of aggressive responses.

### RNA extraction and RT-qPCR

In this study, the expression of the following genes was assessed: vitellogenin (*Am-Vg*); antioxidant-related genes including catalase (*Am-CAT*) and superoxide dismutase (Am-SOD); heat shock proteins (*Am-HSP70* and *Am-HSP90*); antimicrobial peptide genes including apidaecin (*Am-Api*), abaecin (*Am-Aba*), defensin (*Am-Def*), and hymenoptaecin (*Am-Hym*). Actin (*Am-actin*) was used as the reference gene. Total RNA was extracted from whole body of adults using Trizol reagent (Invitrogen, Carlsbad, CA, USA) according to the manufacturer’s instructions. The extracted RNAs were quantified using a spectrophotometer (NanoDrop, Thermo Fisher Scientific). RNA extract (100 ng per reaction) was used for cDNA synthesis with an RT-premix (Intron Biotechnology, Seoul, Korea). Quantitative PCR (qPCR) was performed using SYBR Green Real-Time PCR master mixture (Toyobo, Osaka, Japan) on a Real-Time PCR System (Step One Plus Real-Time PCR System, Applied Biosystems, Singapore). The reaction mixture (20 µL) contained 10 pmol of gene-specific primers (Table S2) used in RT-PCR and 80 ng of cDNA template. After activating Hotstart Taq DNA polymerase at 94 °C for 5 min, the reaction was amplified with 40 cycles of denaturation at 94 °C for 20 s, annealing at a specific temperature depending on primers (Table S2) for 20 s, and extension at 72 °C for 15 s. The target gene expression levels were normalized to those of *Am-actin*, a reference gene. Each treatment was replicated with three independently prepared biological samples. Quantitative analysis was performed using the comparative CT (2^− ΔΔCT^) method (Livak and Schmittgen, 2001)^78^.

### Data analysis

Statistical analyses were performed using GraphPad Prism version 8.2.0 (GraphPad Software, San Diego, CA, USA) and IBM SPSS Statistics version 30 (IBM Corp., Armonk, NY, USA). For survival analysis, Kaplan–Meier survival curves were generated and compared using the log-rank (Mantel–Cox) test. Depending on data distribution, parametric or non-parametric tests were used. ANOVA followed by post hoc Tukey’s test was applied for group comparisons when assumptions were met. For behavioral data, the Mann-Whitney U test was used for score-based comparisons, while the Student’s t-test was applied for total score measurements. Descriptive statistics were expressed as “mean ± standard error” or “median (minimum–maximum).” To assess gene expression data over time and across treatment groups, a two-way mixed-effects ANOVA model was used, considering “time” (days post-treatment) and “treatment” (feeding regime or concentration) as fixed effects and “replicate” as a random effect. When significant interactions were observed, simple effects were analyzed using Bonferroni-corrected pairwise comparisons.

## Supplementary Information

Below is the link to the electronic supplementary material.

Supplementary Material 1

## Data Availability

All data generated or analyzed during this study are included in this published article and its supplementary information file. The genome sequence datasets generated and/or analyzed during the current study are available in the GenBank repository (https://www.ncbi.nlm.nih.gov/genbank) using accession numbers provided in Table S4.
