# Supplementary material for: Behavioral and molecular disruptions in honey bees induced by lithium chloride exposure
Source: Sci Rep. 2025 Oct 27;15:37439. doi: 10.1038/s41598-025-21359-x (PMC12559172; doi:10.1038/s41598-025-21359-x)
Supplement: Supplementary file 1 — Supplementary Material 1 [file 41598_2025_21359_MOESM1_ESM.docx]

**Supplementary Material**

**Table S1.** Analytical measurement parameters

| **Replicates Pump Speed** | **3**  **15 rpm** |
| --- | --- |
| Uptake time | 15 s |
| Rinse time | 30 s |
| Stabilization time | 15 s |
| Read time | 3 s |
| Nebulizer pressure | 240 kPa |
| Wavelength | 610.365 nm |

**Table S2.** Primers used in this study

| **Genes** | **Orientation** | **Sequence (5ʹ - 3ʹ)** | **Uses** | **Annealing temperature (ºC)** |
| --- | --- | --- | --- | --- |
| *β-Actin* | Forward | CTAGCACCATCCACCATGAAA | RT-qPCR | 52.0 |
|  | Reverse | AGGTGGACAAAGAAGCAAGAA |  |  |
| *HSP70* | Forward | GCTGTACAGGCCGCTATTT | RT-qPCR | 52.0 |
|  | Reverse | ACACCACCAGCTGTTTCTATAC |  |  |
| *HSP90* | Forward | TGGTATGACAAAGGCTGATCTT | RT-qPCR | 52.0 |
|  | Reverse | AACCTACACCAAACTGACCTATC |  |  |
| *Vitellogenin* | Forward | GCAGAATACATGGACGGTGT | RT-qPCR | 52.0 |
|  | Reverse | GAACAGTCTTCGGAAGCTTG |  |  |
| *Catalase* | Forward | GTCTTGGCCCAAACAATCTG | RT-qPCR | 52.0 |
|  | Reverse | CATTCTCTAGGCCCACCAAA |  |  |
| *Superoxide dismutase* | Forward | AAGCAGTGTGCGTTCTTCAGGGT | RT-qPCR | 52.0 |
|  | Reverse | TCACGGAATTGGTACTCTCCGGTT |  |  |
| *Abaecin* | Forward | CAGCATTCGCATACGTACCA | RT-qPCR | 52.0 |
|  | Reverse | GACCAGGAAACGTTGGAAAC |  |  |
| *Defensin* | Forward | TGCGCTGCTAACTGTCTCAG | RT-qPCR | 52.0 |
|  | Reverse | AATGGCACTTAACCGAAACG |  |  |
| *Hymenoptaecin* | Forward | CTCTTCTGTGCCGTTGCATA | RT-qPCR | 52.0 |
|  | Reverse | GCGTCTCCTGTCATTCCATT |  |  |
| *Apidaecin* | Forward | TTTTGCCTTAGCAATTCTTGTTG | RT-qPCR | 52.0 |
|  | Reverse | GTAGGTCGAGTAGGCGGATCT |  |  |

**Table S3.** Lithium impact on aggressive behavior in *A. mellifera*. Frequency of individual aggressive behaviors (antennation, mandible opening, chasing, biting, and stinging) in control and LiCl-fed bees during the intruder assay.

| **Variables** | **Groups** | | | | | | **P** |
| --- | --- | --- | --- | --- | --- | --- | --- |
|  | **Negative Control** | | | **Lithium10** | | |  |
|  | **n** | **Mean ± SEM** | **Median (Min.-Max.)** | **n** | **Mean ± SEM** | **Median (Min.-Max.)** |  |
| **Antennation** | 10 |  | 1 (1-2) | 10 |  | 0.5 (0-2) | 0.089 |
| **Mandible Op.** | 10 |  | 2.5 (1-4) | 10 |  | 1 (0-3) | 0.075 |
| **Chasing** | 10 |  | 1.5 (0-3) | 10 |  | 0 (0-2) | 0.019 |
| **Biting** | 10 |  | 3 (2-4) | 10 |  | 2.5 (0-4) | 0.436 |
| **Stinging** | 10 |  | 2 (1-3) | 10 |  | 1 (0-1) | <0.001 |
| **Total Score** | 10 | 32.8 ± 1.81 | 31 (24-42) | 10 | 17.4 ± 2.34 | 18 (3-27) | <0.001 |

**Table S4**. GenBank accession numbers of genes used in this study

| **Gene name** | **Acronym** | **Accession number** |
| --- | --- | --- |
| Vitellogenin | *Am-Vg* | NM_001011578 |
| hymenoptaecin | *Am-Hym* | U15956 |
| defensin | *Am-Def* | U15955 |
| abaecin | *Am-Aba* | NM_001011617 |
| apidaecin | *Am-Api* | X72575.1 |
| Catalase | *Am-CAT* | NM_001178069 |
| superoxide dismutase | *Am-SOD* | NM_001178027 |
| Heat shock protein 70 | *Am-HSP70* | NM_001160072 |
| Heat shock protein 90 | *Am-HSP90* | NM_001160064 |
| Actin | *Am-Actin* | NM_001185146 |


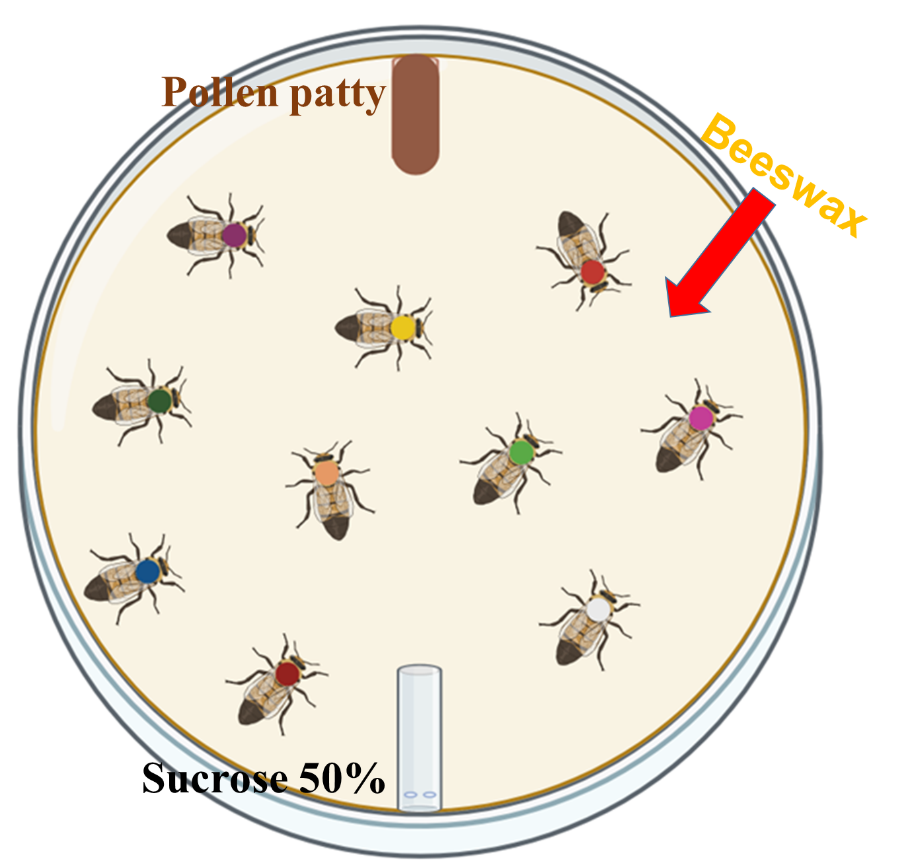


**Fig. S1.** Petri dishes (90 x 20 mm) lined with a beeswax foundation plate to mimic hive conditions for behavioral experiment.
